# Outcomes and complications of conversion THA after internal fixation of proximal femur fractures: a systematic review

**DOI:** 10.1007/s00068-025-02977-6

**Published:** 2025-09-16

**Authors:** Alberto Di Martino, Claudio D’Agostino, Riccardo Poluzzi, Matteo Brunello, Giuseppe Geraci, Francesco Traina, Cesare Faldini

**Affiliations:** 1 Department of Biomedical and Neuromotor Sciences (DIBINEM), University of Bologna, Bologna, Italy; 2 1st Orthopedic and Traumatology Department, IRCCS Rizzoli Orthopedic Institute, Bologna, Italy; 3 Department of Orthopedics and Traumatology and Hip and Knee Arthroplasty and Revisions, IRCCS Rizzoli Orthopedic Institute, Bologna, Italy

**Keywords:** Conversion THA, Revision, Internal fixation, CTHA, Total hip arthroplasty, Complication

## Abstract

**Purpose:**

The goal of this systematic review is to analyse clinical results and complications of conversion total hip arthroplasty (cTHA) after failure of internal fixation (IFix), distinguishing according to the initial fixation method: intramedullary nail, plate/screw systems, and cannulated screws.

**Methods:**

PubMed, EMBASE, and Cochrane database were firstly accessed in April 2025, and lastly checked on July 2025, to identify studies addressing patients who underwent to cTHA after sustaining proximal femoral fractures with subsequent IFix. The PRISMA guidelines were followed, and the quality of studies was assessed. Data were extracted from the identified articles and summarised.

**Results:**

Twelve retrospective studies on 1,260 patients analyzed complications of conversion to total hip arthroplasty (cTHA) after internal fixation of the femur. After fixation with an intramedullary nail, cTHA showed a 6.01% dislocation rate, 3.14% periprosthetic fractures, 2.59% aseptic loosening, and 3.41% periprosthetic infections, with a 3.82% reoperation rate. Fixation with a plate/screw system resulted in fewer complications but a higher incidence of periprosthetic fractures (11.57%). A notably higher dislocation rate of 10.04% was observed following fixation with cannulated screws.

**Conclusion:**

This review confirms that cTHA after IFix of proximal femoral fractures is associated to an elevated rate of perioperative complications, with a higher incidence when cTHA is performed after intramedullary nail fixation; a higher incidence of intraoperative periprosthetic fractures is observed on those patients treated by plate/screws system at first surgery. Unexpectedly, a higher rate of implant dislocations is recorded in those patients undergoing cTHAs after IFix by cannulated screws.

**Supplementary Information:**

The online version contains supplementary material available at 10.1007/s00068-025-02977-6.

## Introduction

Proximal femoral fractures are a common and severe injury affecting both the elderly and the adult population, with 1.6 million cases occurring annually worldwide; this incidence is predicted to increase, with an occurrence of 6.3 million in 2050 [1].

In most cases, treatment is aimed at fracture reduction and bone stock preservation, preferring Internal Fixation (IFix) to total hip arthroplasty (THA) [2]. Currently, a variety of devices are available for the treatment of proximal femur fractures. It is widely agreed that Pauwels Type I intracapsular fractures should be addressed using multiple cannulated screws. However, sliding hip screws and side plate design devices (such as the Femoral Neck System, FNS), as well as hemiarthroplasty, are effective treatment options, particularly for Pauwels Type II and III fracture patterns [3]. On the other hand, extracapsular fractures are best treated with an intramedullary device (short or long cephalo-medullary nail, CMN) or other sliding screw and side plate design devices (e.g., dynamic hip screw, DHS; proximal femur locking compression plates, PF-LCP; dynamic compression plate, DCP; etc.) [3, 4].

Numerous studies compared intramedullary with extramedullary devices; however, inconsistent results were reported; so far, no study demonstrated a clinical superiority of any of these devices in terms of fracture healing [5–8].

IFix procedures at the proximal femur are not exempt from complications, ranging from avascular necrosis (AVN) of the femoral head, malunion or nonunion, and mechanical fixation failure which may ultimately lead to the development of secondary osteoarthritis [3, 4].

Despite the availability of multiple treatment choices for femoral neck fractures, the options for subsequent revision procedures are limited, particularly when dealing with complex proximal femoral anatomical deformities resulting from a failed fracture fixation, bone deficiencies, and in patients with inadequate muscle balance [9]. In those cases, an effective salvage treatment entails the extraction of the implanted device followed by implant of a THA: this surgery is commonly referred to as conversion THA (cTHA) [10–14].

This surgery requires an extended surgical exposure, the management of a weak and osteoporotic bone, and removal of retained hardware before cTHA performance; not last, surgeons and anesthesiologists are required to manage the fragile nature typical of the elderly population [15, 16]. These elements collectively exacerbate the risks associated with cTHA surgery following a previous IFix. Consequently, cTHA has been reasonably compared to revision THAs in terms of blood loss, dislocation rate, duration of hospitalization, and overall costs for the National Health System [17].

**Fig. 1:**
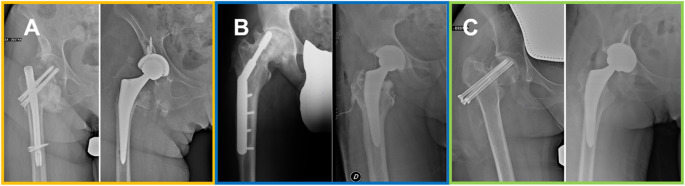
cTHAs performed after intramedullary nail (A-orange), plate/screw system (B-blue) and cannulated screws (C-green) removal

While our systematic review is not the first on this topic, the purpose is to update and refine previous findings considering emerging data reporting on cTHA after IFix failure for proximal femoral fractures, focusing on clinical results and complications; results will be reported separately according to the initial method of fixation: intramedullary nail, plate/screw systems and cannulated screws (Fig. 1).

## Materials and methods

### Eligibility criteria

The PICOS model (Population, Intervention, Comparison, Outcomes, Study design) was used for the present study. The authors included studies that considered patients who underwent IFix for proximal femoral fracture (Population), undergoing a cTHA (Intervention), with or without a comparison group of primary THA controls (Comparison), that reported differences on blood loss, procedure time, average follow-up and Harris Hip Score (HHS) at twelve months, and complications such as hip dislocations, intra- and post-operative fractures, aseptic loosening and periprosthetic joint infection that required or not a revision surgery (Outcomes); Randomized Controlled Trials (RCTs), retrospective or prospective observational studies (Study design) that met the abovementioned PICOS were included; reviews, case reports and poor case series were excluded.

No limits regarding the year of publication were applied. Only articles in English language were considered.

### Search strategy

A systematic review of the available English literature on three large databases (Scopus, Embase and PubMed) was performed in April 2025 and lastly checked on July 2025.

Medical subject headings (MeSH) terms used were “femur fracture,” “nail”, “screw”, “DHS”, “FNS”, “plate” and “conversion”, “THA” and “cTHA”. The following string was used: (femur fracture) AND (nail OR screw OR DHS OR FNS OR PLATE) AND (conversion OR THA OR CTHA).

**Fig. 2:**
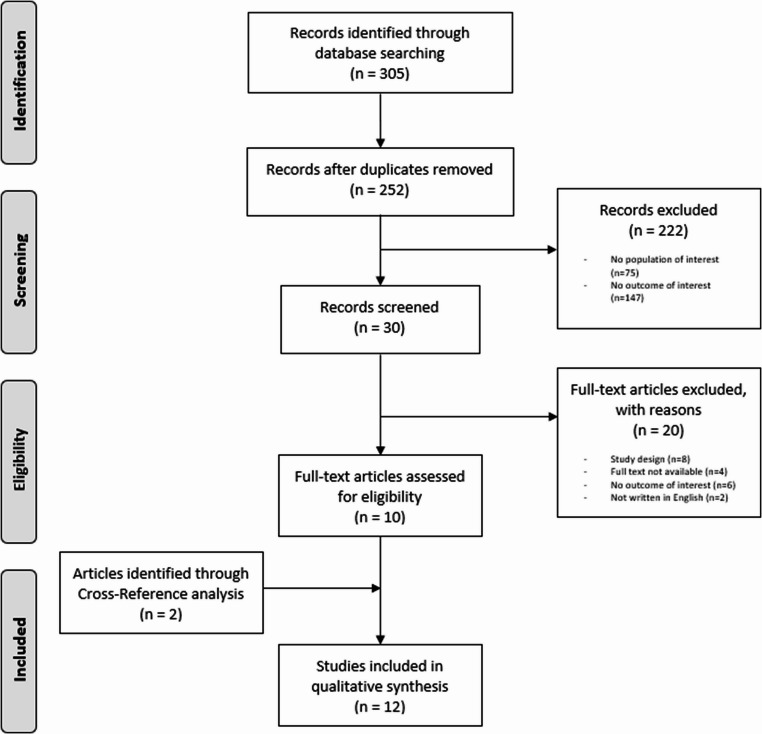
Search strategy according to the preferred reporting items for systematic reviews and meta-analyses (PRISMA) statement

Additional articles were found through a cross-reference search of eligible studies. Two authors (CD and RP) independently screened all potentially relevant titles and abstracts, and any disagreement was solved by the senior authors (ADM and CF). The Preferred Reporting Items for Systematic reviews and Meta-Analyses recommendations were followed during this review [18](Fig. 2).

Two authors (GG and FT) independently evaluated the methodological quality of the included studies using the NIH tool [18]. If any disagreement arose, it was resolved by a discussion with a third senior author (ADM). Reference data, populations and outcomes were extracted from the articles into pre-specified tables using a standardized data extraction procedure by two of the authors (RP and MB). They extracted information on studies’ general characteristics (including design and primary outcomes), number of patients (population), interventions (cTHA after nail, screws and/or plate and screws), comparator (none), and the summary of main outcomes.

### Statistical analysis

Descriptive statistics were used to summarize the data, presented as median and mean with standard deviation (SD) for continuous variables and as frequency with percentage (%) for categorical variables. Descriptive statistics were reported when data to pool were evaluated insufficient, or when substantial clinical heterogeneity was observed in terms of population characteristics, intervention types, or outcome assessment methods.

## Results

Twelve studies reporting on a total of 1,260 patients were included; these were all retrospective case series in which instrumentation removal and cTHA were performed during the same surgery; the populations presented in the selected studies were mostly concordant in age, ASA score, and BMI; 64.8% (817 cases) were females and 443 were males. The average age at cTHA was 70.58 years (± 5,31 years), while the average follow-up time was 48.57 months (± 4.3 months). The overall quality of the included studies was judged as “good” (Supplementary Table 1).

### Conversion-THAs after intramedullary nailing

We pooled data from 7 studies [1, 9, 19–23], resulting in a cohort of 733 cTHAs. The average BMI was 26.3 Kg/m^2^ (± 3.5 Kg/m^2^), the median ASA score was 2 (range 1–3), the average intraoperative blood loss was 858.6 mL, and the average surgical time was 116 min (Table 1). The average Harris Hip Score (HHS) at 12 months was 84.19 ± 3.12 (Table 2). Regarding surgical complications, implant dislocations occurred in 44/733 cTHAs (6.01%), 23 patients had an intra- or post-operative periprosthetic fracture (3.14%), 19 presented aseptic loosening (2.59%) and 25 developed a periprosthetic joint infection (3.51%). A reoperation was necessary in 28 cTHAs (3.82%) (Table 3).

**Table 1:** Groups characteristics at cTHA surgery

Groups characteristics at cTHA surgery	Intramedullary nail	Plate and screws	Cannulated screws
Pt. n°	733	268	259
BMI (kg/m²)	26.4 (range 23.2–29.5) ^19–21^	25.5 (range 23.2–28.9) ^19,21,24,26^	26 (range 26–26.1) ^26,28^
ASA Score	2 (range 1–3) ^19,21,23^	2 (range 2–3) ^19,21,23,24,26^	2 (range 1–2) ^23,26,27^
Mean Blood Loss (ml)	858.6 ^19^	911.65 ± 395 ^19,23–25^	516.7 ^25^
Mean Procedure Time (min)	116 ^19,23^	122 ^19,23–26^	97 ^23,26^

**Table 2:** Groups characteristics at cTHA surgery

Clinical Outcome of cTHA after	Authors	Average follow-up (months)	HHS at 12months
Intramedullary Nail	Selim et al. 2025	48	87
	Jin et al. 2021	N/A	85.4
	Godoy et al. 2021	63.6	86
	Yu et al. 2020	65.15	85.62
	Smith et al. 2019	N/A	N/A
	Zeng et al. 2017	47.92	82.54
	Pui et al. 2013	35	78.6
	**Tot.**	**51.93** ± 12.54	**84.19** (78.6–87)
Plate and Screws	Selim et al. 2025	48	86
	Jin et al. 2021	N/A	85.4
	Morice et al. 2018	42	78.7
	Zeng et al. 2017	48.1	81.9
	Pui et al. 2013	37	83.6
	Winemaker 2006	N/A	73.9
	Zhang et al. 2004	N/A	79.9
	**Tot.**	**43.78** ± 5.34	**81.34** (73.9–86)
Cannulated Screws	Selim et al. 2025	48	87
	Hernandez et al. 2017	24	85
	McKinley et al. 2010	78	N/A
	Winemaker et al. 2006	N/A	82.6
	**Tot.**	**50** ± 27.06	**84.87** (82.6–87)

**Table 3:** Clinical outcomes

Complications of cTHA after Intramedullary Nail						
Authors	Hip dislocations	Periprosthetic fracture [Intra-Op]	Aseptic loosening	Periprosthetic joint infection	Revision	Tot. (%)
Selim et al. 2025	2	1 (3)	0	0	1	7
Jin et al. 2021	1	0	0	0	1	2
Godoy et al. 2021	2	0	0	0	0	2
Yu et al. 2020	6	13	18	0	14	51
Smith et al. 2019	30	N/A	N/A	22	12	64
Zeng et al. 2017	1	3	0	2	0	6
Pui et al. 2013	2	3	1	1	N/A	7
Pt. 733 (100%)	**44** **(6.01%)**	**23 (3.14%)** **[3 (0**,**41%)]**	**19** **(2.59%)**	**25** **(3.41%)**	**28** **(3.82%)**	**139 (18.96%)**

### Conversion-THAs after plate and screws

With data extrapolated from seven studies [19, 21–26] a cohort of 268 cTHAs were performed after removal of plate/screw system; in this group were included the different systems based on sliding hip screw and side plate design (i.e., DHS, FNS, etc.). The mean BMI was 25.5 Kg/m^2^ (± 2.5 Kg/m^2^), the median ASA score was 2 (range 2–3), the average blood loss was 911.65mL (± 395.88mL) and the average surgical time was 122 min (Table 1). Average HHS at 12 months was 81.34 ± 4.24 (Table 2). As regards surgical complications, implant dislocation occurred in 7 patients (2.61%), intra-operative periprosthetic fracture occurred in 12 cTHAs (4.48%), 19 patients (7.09%) experienced a post-operative periprosthetic fracture, 2 patients (0.75%) developed aseptic loosening and 3 patients (1.12%) developed a periprosthetic joint infection. Revision surgery was required in 5 cases (1.87%) (Table 4).

**Table 4:** Complications of cTHA after plate and screws

Complications of cTHA after Plate and Screws						
Authors	Hip dislocations	Periprosthetic fracture [Intra-Op]	Aseptic loosening	Periprosthetic joint infection	Revision	Tot. (%)
Selim et al. 2025	0	0 [2]	0	0	0	2
Jin et al. 2021	0	0	0	0	0	0
Morice et al. 2018	1	3	0	0	3	7
Zeng et al. 2017	3	11	1	2	N/A	17
Pui et al. 2013	0	4	0	1	0	5
Winemaker et al. 2006	N/A	1 [3]	1	N/A	2	7
Zhang et al. 2004	3	0 [7]	0	0	0	10
Pt. 268 (100%)	**7 (2.61%)**	**31 (11.57%) [12 (4.48%)]**	**2 (0.75%)**	**3 (1.12%)**	**5 (1.87%)**	**48 (17.91%)**

### Conversion-THAs after cannulated screws

We pooled data from three studies [23, 26–28] with a cohort of 259 cTHA implants. The average BMI was 26.0 ± 0.1 Kg/m^2^, median ASA score was 2 (1–2), the average intraoperative blood loss was 516.7mL, and the average procedure time was 97 min (Table 1). Average HHS at 12 months was 84.87 ± 2.20 (Table 2). Hip dislocation occurred in 26 patients (10.04%), intra-operative periprosthetic fracture occurred during 9 procedures (3.47%), and 2 patients (0.77%) had post-operative periprosthetic fracture. Nine patients (3.47%) had aseptic loosening and 10 (3.86%) developed a periprosthetic joint infection. A revision surgery was necessary in 18 cases (6.95%) (Table 5).

**Table 5:** Complications of cTHA after cannulated screws

Complications of cTHA after Cannulated Screws						
Authors	Hip dislocations	Periprosthetic fracture [Intra-Op]	Aseptic loosening	Periprosthetic joint infection	Revision	Tot. (%)
Selim et al. 2025	3	0	0	0	1	4
Hernandez et al. 2017	2	0 [2]	1	1	4	10
McKinley et al. 2010	21	2 [3]	8	8	12	54
Winemaker et al. 2006	0	0 [4]	0	1	1	6
Pt. 259 (100%)	**26 ** **(10.04%)**	**11 (4.25%)** **[9 (3.47%)]**	**9 ** **(3.47%)**	**10** **(3.86%)**	**18** **(6.95%)**	**74 (28.57%)**

As a secondary outcome, complications were pooled based on the surgical approach used during cTHA. In most of the included studies, the approach was either unspecified, or not related to the single patient; therefore, maintaining the division into three groups according to the instrumentation used, data on post-operative complications from six studies [1, 19, 21, 23, 24, 27] on cTHAs performed by posterolateral (PL) approach were considered. From these 6 studies, 135 cTHAs after intramedullary nailing were performed with the PL approach [1, 19, 21, 23]; implant dislocation occurred in 6 cases (4.4%), intra- or post-operative periprosthetic fracture in 3 (2.2%), and 2 (1.5%) patients developed a periprosthetic joint infection. Reoperation was necessary in 3 of these 135 patients (2.2%).

From a cohort of 146 cTHAs performed after plate and screws removal by PL approach [19, 21, 23, 27], hip dislocation occurred in 6 patients (6.1%), periprosthetic fracture occurred intraoperatively during 20 cTHAs (13.7%), 1 patient developed aseptic loosening and 2 (1.14%) had a periprosthetic joint infection. Only two studies [23, 24] evaluated cTHAs after cannulated screws removal by PL approach. In a population of 183 cTHAs, hip dislocation occurred in 5 patients (2.7%), 7 (3.8%) periprosthetic fractures occurred, 8 patients (3.4%) presented aseptic loosening and 8 (3.4%) developed a periprosthetic joint infection. Revision surgery was required in 13 cases (7.1%) (Table 6).

**Table 6:** Surgical complications of cTHA in PL approach

Surgical complications of cTHA in Posterolateral approach						
	Pt. *n*°	Hip dislocation	Periprosthetic fracture	Aseptic loosening	Periprosthetic joint infection	Revision
Intramedullary Nail	135	6 (4.4%)	3 (2,2%)	0 (0%)	2 (1.5%)	3 (2.2%)
Plate and Screws	146	6 (4.1%)	20 (13.7%)	1 (0.7%)	2 (1.4%)	0 (0%)
Cannulated Screws	183	5 (2.7%)	7 (3.8%)	8 (3.4%)	8 (3.4%)	13 (7.1%)

## Discussion

This review focuses on a very topical issue. With the increase in the average age of the general population, the number of fractures at the proximal femur is steadily increasing as well as life expectancy after IFix for these fractures. This elderly population usually presents a condition of frailty, characterized by a state of loss of physical reserve and decreased ability to withstand physiological and homeostatic stressors [29], with clinical and functional decline. Frailty places older patients at increased likelihood of biological and mechanical complications of IFix surgery, requiring revision surgery. The effectiveness of cTHA as a salvage procedure for the failure of IFix of proximal femur fractures has already been reported [2, 30]. However, this is a challenging procedure associated with longer surgical times, increased intraoperative bleeding, and higher complication rates compared to primary THAs [2, 30, 31].

Aim of this study was to systematically review current literature and compare clinical outcomes, complication rates, and procedural difficulties among patients initially treated with different open reduction and IFix strategies.

Our study was not exempt of limitations. Firstly, the recovery of data from the selected studies which were all retrospective and reporting on small populations. Another limitation was the need to compare data that lacked uniformity, as there were instances where it was not feasible to differentiate between data associated with different patterns of fracture, cemented and uncemented THA implants, different approaches, or the primary fixation systems.

Regarding clinical and functional outcomes assessed through the HHS, these were consistent across the reviewed studies and were not influenced by the previous fixation devices [19, 21–23, 26]. Conversely, the length of surgery and intraoperative bleeding averaged higher values in cTHA when compared to primary THA, Winemaker et al. [26] describe an average operating time of 76.7 min and an average bleeding of 406.5 mL in primary THA. Similarly, Brunello et al. [32] described an average surgery time of 78.8 min in primary THA performed by direct anterior access and a mean 74 min for the posterolateral approach. Instead, our study reported an average operating time of 100.53 min and an average blood loss of 762.3 mL. These differences were primarily attributed to the removal of instrumentation during cTHA.

As highlighted in the study by Selim et al. [23], conversion procedures following CMN were associated with the longest operative times, with the authors reporting a substantial 45-minute increase in duration compared to cases converted after cannulated screw fixation. Moreover, our study showed an even greater prolongation of operative time in cases requiring the removal of plate and screw constructs, further emphasizing the technical complexity and surgical challenges associated with hardware extraction during cTHA.

The analysis of cTHA after CMN failure reveals complications such as postoperative dislocation and periprosthetic infection, with a notable incidence of dislocation reaching 8.1% within two years in the report by Smith et al. [1]. The use of intramedullary devices like CMN is associated to higher soft tissue damage, in particular at the gluteus medius muscle tendon, possibly leading to an increased risk of dislocation [33]. Achieving proper femoral anteversion is emphasized to minimize complications’ rate and to enhance patient’s satisfaction after CMN failure [9, 34, 35].

Less frequent complications in these patients include aseptic mobilization and periprosthetic fractures. Yu et al. [20] noticed a higher rate of aseptic loosening in patients treated with uncemented rather than cemented THA. The choice between cemented and uncemented implants for this procedure remains unclear due to the lack of comparative studies [36–39]. However, uncemented revision stem fixation could be beneficial for patients if performed by experienced surgeons, particularly in younger and non-osteoporotic patients [36–39], while cemented cTHA should be reserved for older individuals with compromised bone stock [23].

The relatively higher incidence of infections observed in the conversion group can be attributed to several factors, including the presence of hardware, the requirement for extensive surgical exposure and prolonged surgical time, but definitely it reflects the increased comorbidities usually associated to this patients’ population; however, in suspected patients it is appropriate to investigate and exclude infections as the underlying cause of fixation failure of CMN devices before cTHA performance [1, 40].

In the plate/screw group, periprosthetic fractures and postoperative dislocations were the most significant complications. The association between periprosthetic fractures and previous IFix by DHS has already been reported by several studies [21, 25]. The biomechanical impact of stress shielding by the side plate of DHS implants leads to lower mechanical strength at the lateral cortex [41]. These events are less frequently encountered when intramedullary nail implants are used.

Unexpectedly, in patients undergoing cTHA after cannulated screw fixation, the most frequent complication was hip dislocation (12.57%) followed by periprosthetic joint infection (PJI, 5.46%). McKinley et al. [27] reported a high dislocation rate of 19.6% and a PJI rate of 7.5%, attributing these outcomes to the particular frailty of the treated patients and the limited expertise of the surgical team. These outcomes are in contrast with other data collected in the current review [15, 26]. Conversely, Hernandez et al. [28], reported a low dislocation incidence (3%), attributing it to the use of large diameter heads. Hernandez and colleagues studied cTHA after cannulated screw IFix for femoral neck fractures, reporting a low periprosthetic fracture rate, crediting careful surgical strategies, especially leaving screws in place until hip dislocation.

We could not address the question if some complications could be more frequent when a peculiar surgical approach is performed, because only data from studies on PL approach were available, being it the most used approach when CMN or plate implants are on site at cTHA; on the other side, PL approach has an intrinsic risk of THA implant dislocation, particularly in patients with conditions of frailty and osteosarcopenia [2, 28]. It could be interesting to evaluate the role of other approaches including the direct anterior approach and direct lateral approach for cTHA in terms of dislocation rate, seen the lack of comparative studies presenting specific data for cTHAs [42].

## Conclusion

This review confirms that cTHA after IFix of proximal femur fractures is associated to an elevated rate of complications. Our subgroup analysis identified a higher number of complications in cTHAs performed after intramedullary nail fixation, except for a higher incidence of intraoperative periprosthetic fractures in the plate/screw group and a higher rate of hip dislocations in patients undergoing cTHA after cannulated screws.

Patients should be informed that salvage cTHA after failure of IFix is a procedure at risk of early complications, higher compared to primary THAs; moreover, despite an improvement of HHS after surgery, hip function may be poorer compared to primary replacements.

These findings are of critical importance because of the increased rate of cTHA expected in the next future; these data may recalibrate patients’ expectations and improve doctor-patient relationship, eventually decreasing the rate of litigations after cTHA surgery.

## Supplementary Information

Below is the link to the electronic supplementary material.

ESM 1 (DOCX 18.0 KB)

## Data Availability

No datasets were generated or analysed during the current study.
