# Supplementary material for: Outcomes and complications of conversion THA after internal fixation of proximal femur fractures: a systematic review
Source: Eur J Trauma Emerg Surg. 2025 Sep 16;51(1):293. doi: 10.1007/s00068-025-02977-6 (PMC12441094; doi:10.1007/s00068-025-02977-6)
Supplement: Supplementary file 1 — (DOCX 18.0 KB) [file 68_2025_2977_MOESM1_ESM.docx]

| **Studies** | **Was the study question or objective clearly stated?** | **Was the study population clearly and fully described, including a case definition?** | **Were the cases consecutive?** | **Were the subjects comparable?** | **Was the intervention clearly described?** | **Were the outcome measures clearly defined, valid, reliable, and implemented consistently across all study participants?** | **Was the length of follow-up adequate?** | **Were the statistical methods well-described?** | **Were the results well-described?** | **Quality summary** |
| --- | --- | --- | --- | --- | --- | --- | --- | --- | --- | --- |
| **Selim et al. 2025** | **✔** | **✔** | **✔** | **✔** | **✔** | **✔** | **✗** | **✔** | **✔** | **2** |
| **Jin** **et al. 2021** | **✔** | **✔** | **✔** | **✔** | **✔** | **✔** | **✗** | **✔** | **✔** | **2** |
| **Godoy et al. 2021** | **✔** | **✔** | **✔** | **✔** | **✔** | **✗** | **✔** | **✔** | **✗** | **2** |
| **Yu** **et al. 2020** | **✔** | **✔** | **✔** | **✗** | **✔** | **✔** | **✔** | **✔** | **✔** | **2** |
| **Smith et al. 2019** | **✔** | **✔** | **✔** | **✔** | **✔** | **✗** | **✗** | **✔** | **✔** | **2** |
| **Morice et al. 2018** | **✔** | **✔** | **✔** | **✔** | **✔** | **✔** | **✔** | **✔** | **✔** | **2** |
| **Zeng et al. 2017** | **✔** | **✔** | **✔** | **✔** | **✔** | **✔** | **✗** | **✔** | **✔** | **2** |
| **Hernandez et al. 2017** | **✔** | **✔** | **✔** | **✔** | **✗** | **✔** | **✔** | **✔** | **✔** | **2** |
| **Pui et al. 2013** | **✔** | **✗** | **✔** | **✔** | **✔** | **✔** | **✗** | **✔** | **✔** | **2** |
| **McKinley et al. 2010** | **✔** | **✔** | **✔** | **✔** | **✔** | **✗** | **✔** | **✔** | **✗** | **2** |
| **Winemaker et al. 2006** | **✔** | **✔** | **✔** | **✔** | **✔** | **✗** | **✗** | **✔** | **✔** | **2** |
| **Zhang et al. 2004** | **✔** | **✔** | **✔** | **✔** | **✔** | **✗** | **✔** | **✔** | **✗** | **2** |

***Supplementary table 1:*** *Quality was rated as 0 for poor (0–3 out of 9 questions), 1 for fair (4–6 out of 9 questions), or 2 for good (7–9 out of 9 questions); NA: not applicable, NR: not reported.*
